# Metrology and Standards Needs for Some Categories of Medical Devices

**DOI:** 10.6028/jres.113.009

**Published:** 2008-04-01

**Authors:** J. C. Chiao, Julian M. Goldman, David A. Heck, Peter Kazanzides, William J. Peine, James B. Stiehl, Dwight Yen, Nicholas G. Dagalakis

**Affiliations:** University of Texas at Arlington, Arlington, TX 76019; Massachusetts General Hospital, Boston, MA 02114; Baylor Health Care System, Dallas, TX 75206; Johns Hopkins University, Baltimore, MD 21218; Purdue University, West Lafayette, IN 47907; Columbia St Mary’s Hospital, Milwaukee, WI 53211; Food and Drug Administration, Rockville, MD 20850; National Institute of Standards and Technology^1^, Gaithersburg, MD 20899-8230

**Keywords:** computer assisted surgical navigation, medical devices, networked medical device interoperability, neurostimulation implants, surgical robots, surgical simulation systems

## Abstract

With rapid advances in meso-, micro- and nano-scale technology devices and electronics, a new generation of advanced medical devices is emerging, which promises medical treatment that is less invasive and more accurate, automated, and effective. We examined the technological and economic status of five categories of medical devices. A set of metrology needs is identified for each of these categories and suggestions are made to address them.

## 1. Introduction

Members of the staff from the Food and Drug Administration (FDA), the National Cancer Institute (NCI), the University of Texas at Arlington (UTA), the National Institute of Standards and Technology (NIST), and ASTM International, in cooperation with other government agencies, private industries, and universities, agreed to collaborate in order to document and prioritize the measurement and measurement-related standards needs of a few categories of medical devices. The ultimate goal of this identification initiative is to help NIST direct resources, or to create partnerships, to provide solutions to these needs (http://usms.nist.gov).

Discoveries and technology developments during the 20th century made possible the development of a significant number of medical devices that have radically transformed the way medical care is delivered today. These include computed axial tomography (CAT) in 1972, magnetic resonance imaging (MRI) in 1977, positron emission tomography (PET) in 1976, implantable defibrillators in 1980, artificial hearts in 1982, cochlear implants in 1985, Lasik eye surgery in 1990, computer assisted surgery systems in 1986, surgical robots in 1990, and diagnostic imaging endoscopy capsules in 2001. Despite the tremendous progress, many problems exist in performance, standards, sensors, and reliability.

A workshop was held on November 14–17, 2006, at the Hyatt Regency Atlanta, Georgia, as part of the ASTM Medical and Surgical Materials and Devices F04 committee week meeting, to bring together experts in the fields of Medical Devices metrology and standards in order to discuss and document the metrology and standards needs of a few categories of medical devices. This workshop did not cover medical imaging as a biomarker, or active implantable medical devices reliability, which have been the subjects of other NIST U.S. Measurement System (USMS) workshops.

The scope of this workshop focused on the metrology and standards needs of the following categories of medical devices:

- Computer assisted navigation and surgery
- Surgical robots (mostly manual control mode)
- Surgical robots and phantom (artifact) devices
- Stimulation devices
- Drug-delivery and physiologic monitoring devices.

Workshop participants addressed the following questions: What technological innovations are at stake? What is the economic significance of the innovations? What technical barriers to the innovations impede progress to the marketplace? At what stages of innovation do the technical barriers appear? What parts of the technical barriers are measurement science or standards development? What are the potential solutions to the measurement and standards development problems? Who are potential providers of solutions? Are there critical roles for agencies of the federal government?

Guest speakers were invited for each medical device category subject listed above. The guest speakers started with a brief review of the subjects and then facilitated discussions with the workshop attendees.

## 2. Computer Assisted Surgical Navigation

### 2.1 Technological Innovation at Stake

Computer assisted surgical navigation (CAS) is an emerging technology [1], [2]. The market for CAS systems inside an operating room has evolved significantly from an athlete body tracking sensor system, which used two lateral effect photodiode camera tracking sensors, to the modern CAS systems, which use two or three charge coupled device (CCD) cameras with active light emitting diode (LED) targets or passive sphere targets illuminated by infrared light. People have also experimented with electromagnetic tracking sensors, with electrical coil targets and other technologies. Although these types of targets do not require line of sight with the sensor and thus can operate inside the human body, they are susceptible to interference from electromagnetic waves reflected by metal surfaces inside the operating room. Computer assisted surgery systems consist of tracking camera sensors, tracking markers (targets), a computer, and other relevant electronics. During an operation the markers are attached to bones, surgical tools and implants. The three dimensional position of the markers in space is determined with respect to a reference frame and, based on that information, the position and orientation of tools, bones and implants are calculated and used to generate useful surgery information. Computer assisted craniofacial and orthopaedic operations [3], [4], [5], have evolved as two of the most important applications of CAS.

This technology is being used in support of health care services that rely on four dimensional (three dimensions plus time) positioning of instrumentation and prosthetic components within a human reference frame. CAS promises to advance our understanding of optimum device positioning, to improve surgeons’ spatial orientation and to reduce positioning errors that may risk repeat surgeries.

Current CAS devices are provided without clinical traceability characteristics or process capabilities. Testing has revealed variation in performance. Variation appears to depend on multiple factors, including the technology employed, the vendors’ implementation, the user, the specific surgical procedure and the degree(s) of freedom being evaluated. Standardized approaches to the evaluation of the technology employed, usage and reporting of intra-operative findings do not exist. These limitations impede innovation, ongoing device development, marketing, and knowledge development.

### 2.2 Economic Significance

According to data from a Swedish total hip replacement study [6], 8.8 % of revision hip surgery could be attributed to malpositioning of the implant. This includes dislocation (5.8 %) and technical error (3.0 %). A revision orthopaedic surgery is significantly more risky and painful than the original operation.

Based on the Agency for Healthcare Research and Quality (AHRQ)—2004 Health Care Utilization Project (HCUP) [7] data, 431,485 primary and 35,048 revision total knee replacement procedures were performed in the United States. A total of 225,900 primary and 37,115 revision hip replacements were also performed. These procedures represent approximately $26 billion in annual health care system charges and 1,454 deaths per year (weighted in-hospital mortality = 0.199 %). Preliminary results would suggest that a significant proportion of revision procedures performed may be eliminated through the improved prosthetic positioning associated with the use of CAS at the time of surgery.

### 2.3 Technical Barrier

Performance studies of CAS tracking systems have shown that they have positioning errors, which increase as the surgeon approaches the edge of the workspace [8], [9], [10], [11]. Following is a list of possible sources of CAS tracking systems errors:

- Camera optics
- Detector irregularities
- Target operating conditions, like temperature, nonuniform radiation field, distance from the camera sensors, etc.
- Camera position and orientation determination with respect to the tracking sensor system reference coordinate frame
- Sampling rate frequency of multiple targets.

The image generated by each target on the camera tracking sensor is usually an irregular blob with nonuniform intensity distribution. It falls to the controller of each tracking system to decide assignation of *x,y* coordinates to this type of image. A simple rotation of the target, with no position change, could alter the value of the measured *x,y* coordinates. In the case of slow sampling rate tracking systems, the target might move while its position is being sampled.

Traceable metrological standards, validated testing protocols that simulate the end-use environment, standardized reporting, and electronic health reporting standards are needed. The technical barriers must be addressed to facilitate innovation, ongoing development, traceability, process capability evaluation, cost-effectiveness determination and continuation of the movement of this technology from the laboratory to the operating room.

Phantoms (artifacts) that support traceability to standards organizations are needed to confirm basic metrology. The development of standardized phantoms and testing protocols will allow development of metrics to establish validity and facilitate comparison between systems. Phantoms are required that replicate “standard” and “outlier patients.” From the geometric perspective, range validation across the wide range of patient sizes (short, normal, tall) and soft tissue perspectives (aesthetic, normal, morbidly obese) is needed. Standardized and representative anatomic referencing landmarks (fiducials) would facilitate process capability determination in the laboratory. In support of radiographic evaluation, the phantoms will need to have x-ray absorption characteristics comparable to the range of human presentations. Standardized test environments and protocols that replicate the operating room environment including devices that may introduce error through mechanisms such as electromagnetic interference are needed. Clinical investigations to refine our understanding of device position on clinical outcomes require systems that can support large scale data retrieval in a standardized fashion. The intraoperative measurement protocols must address anatomic site, referencing approach, component positioning, navigational technologies employed, prosthetic technologies, metadata, and calibration status such that process capabilities can be established.

## 3. Accuracy Assessment of Surgical Robots

### 3.1 Technological Innovation at Stake

The use of robots to assist with surgical procedures is expected to increase in the coming years and has the potential to dramatically improve patient outcomes. Currently, there are two types of robotic systems: (1) teleoperated or “remote control” robots that enhance a surgeon’s visualization and dexterity during minimally invasive procedures, and (2) surgical computer-aided design/computer-aided manufacturing (CAD/CAM) robots that accurately execute a series of motions based on a preoperative plan. The “daVinci System” from Intuitive Surgical is the only commercially available teleoperated system and has been used for urology, cardiology, pediatrics, obstetrics/gynecology, and general surgery. CAD/CAM style robots are predominantly used for neurosurgery and orthopaedics and combine preoperative medical imaging (CT, MRI) with the accuracy of the robot to achieve optimal intra-operative positioning (e.g., preparing the femur or tibia for precise implantation of hip or knee prostheses, as performed by ROBODOC [12]). Currently, CAD/CAM robots are not as prevalent as teleoperated robots, but both technologies show promise for future commercialization.

More recently a new generation of compact, bone-mounted or hand-held robots is emerging. Examples include the MiniAture Robot for Surgical procedures (MARS, commercialized as SpineAssist) [13], Mini Bone-Attached Robotic System (MBARS) [14], and Praxiteles [15]. The objective is to give better instruments/power tools to the surgeon and to couple him/her with information generated by imaging or other type of sensors.

### 3.2 Economic Significance

According to Business Communications Company [16], the total worldwide market for medical robotics and computer-assisted surgery (MRCAS) devices and equipment is expected to be $1.3 billion in 2006 and $5.7 billion by 2011, with an average annual growth rate (AAGR) of 34.7 %. Surgical robots are the fastest growing U.S. market segment within MRCAS, with a projected AAGR of over 43 % between 2006 and 2011. In addition, surgical robots have the potential to have a significant impact on the overall healthcare delivery system because minimally invasive surgery provides large cost savings over traditional surgery and robotic surgery can reduce inventory and sterilization costs by eliminating the need for other instruments. Neurosurgical applications accounted for approximately 40 % of the MRCAS market in 2005. Endoscopic applications are the fastest growing segment of the MRCAS market and are expected to reach 45 % of the market by 2011.

### 3.3 Technical Barrier and Potential Solutions

The positional accuracy of a surgical robot is critical to maintain patient safety and achieve the best clinical results. Many applications require sub-millimeter positioning accuracy and a few degrees angular orientation accuracy. Position and orientation measurements are difficult to measure routinely during an actual operation, especially when minimal access to the operated part is required. Validating this accuracy is difficult due to the complexity of recreating the operating room conditions in a laboratory setting. For example, orthopaedic robots rely on some method for aligning the robot to the target bone and often require static and/or dynamic localization of anatomic points (e.g., static localization of points on the bone surface and/or dynamic localization of the center of joint rotation). For teleoperated robots, validation of positional accuracy is important to allow these systems to integrate with medical imaging and to facilitate the design of new smaller, cheaper robotic systems.

A major challenge is the creation of measurement objects (artifacts) that sufficiently reproduce the clinical scenario. This requirement may lead to artifacts with features that are difficult to measure with existing devices (i.e., precisely measuring the tip position of a divot, center of rotation of a joint.) For teleoperated robots, new force and position sensors are needed to implement and validate haptic feedback and advanced control algorithms.

Artifacts and measurement systems are needed that are more representative of human anatomy and clinical situations. For example, if the robotic system requires dynamic localization of the center of a joint, the artifact should include an element that mimics the actual motion of the joint. In addition, artifacts are needed that reproduce motions caused by physiological processes such as breathing or blood flow, or fixturing limitations (e.g., not being able to clamp due to space constraints, small incisions, or potential damage to soft tissues and organs.)

The safety of surgical robots can be degraded by poor positioning accuracy. Risk management and fail-safe and fault tolerant design can address some of the robot safety problems. Elimination of single points of failure is possible with the use of redundant sensors, sensors to detect failures in other components, redundant software, periodic diagnostic testing, watchdogs, and independent safety systems.

## 4. Validation of Surgical Simulation Systems

### 4.1 Technological Innovation at Stake

Surgical training has traditionally been accomplished through clinical observation and mentoring from an expert surgeon at the patient's side in the operating room. New technology, such as teleoperated surgical robotics, impede this process and introduce challenges for both the student and teacher, ultimately decreasing patient safety. Virtual reality based surgical simulation trainers have been cited as a solution, allowing surgical residents and fellows to practice and learn at their own pace in a safe, reduced stress environment. Since the induced instrument motions are captured in software, performance metrics can be implemented to evaluate the skill of the students and chart their improvement over time [17]. In addition, experienced surgeons can be certified and practice difficult clinical situations. Ultimately, surgical simulators will be combined with patient specific anatomic models created from preoperative images to allow surgeons to realistically practice a procedure before the actual case.

Experience has shown that robotic surgery requires a minimum of 10 practice cases. During the following 11 to 100 cases, patient specific learning takes place, which continues for up to more than 700 cases.

### 4.2 Economic Significance

Medical errors are a significant problem and lead to increases in the cost of healthcare delivery. Medical robotics and computer-assisted surgery (MRCAS) have the potential to reduce errors and improve patient outcomes. To date, adoption of surgical robotics has been slowed due to the complexity of the current devices and a lack of training techniques. Improved training through simulation will certainly increase these numbers.

### 4.3 Technical Barrier and Potential Solutions

Surgical simulators are multidisciplinary systems and require specialized haptic interfaces, stereo visualization, advanced graphics, and accurate modeling of deformable tissues in real time. To be an effective training tool, the simulator must adequately recreate the feel and performance of actual surgery. The level of performance depends on the specific training objectives. Training basic skills may be possible with simple block-like objects, while teaching proper technique for suturing soft tissues may require very realistic tissue motions. The challenge therefore lies in understanding what aspects of the simulation are important and determining performance requirements and metrics for validation. For example, can a surgical simulator be used to measure the difference between proper and improper robot performance? Can a surgical simulator be used to determine common surgical mistakes and feedback improvements into future robot designs?

Measurement devices are needed to understand how faithful a surgical simulation is to live surgery. Improper surgical simulator training can lead to safety hazards. Key parameters include measurement of real tissue deformations (bulk stiffness and local shape changes), contact interactions with instruments, realistic coloring and texturing of tissues, and force feedback. The haptic interfaces used to interact with the simulator must also be measured and validated. In addition, validated skill evaluation metrics are needed to quantify training level.

New measurement technology and testing protocols are needed to quantify deformations of a variety of real tissues during typical surgical manipulations. Comparisons to the simulated deformations would then be possible. Measurement of actual robot or instrument motions is also needed to validate the faithfulness of the simulation to the real system performance.

## 5. Neurostimulation Implants

### 5.1 Technological Innovation at Stake

Neurostimulation is the stimulation of neural tissues with relatively low electrical impulses by electrodes placed in the vicinity of targeted neurons. Neurostimulation has been used or studied for treatment of chronic pain, essential tremors, Parkinson’s disease, dystonia, obsessive-compulsive disorder, depression, tinnitus, epilepsy, respiratory support, pain due to malignancies spasticity, amyotrophic lateral sclerosis, Huntington’s disease, gastroparesis, irritable bowel syndrome, profound deafness (cochlear implant), headaches, traumatic brain injury, angina pain, peripheral vascular disease pain, pelvic pain, incontinence, and sexual dysfunction, with deep brain, occipital nerve, pulmonary, gastric, vagus nerve, peripheral nerve, spinal cord, or sacral nerve stimulation. Neurostimulation has provided a safe and effective treatment method for neurological disorders. As an example, neurostimulation can be used to reduce chronic pain by blocking transmission of pain messages to one's brain. Patients may feel a mild tingling sensation instead of pain with stimulation electrodes on their spinal cords delivering alternating currents (AC) into the tissues [18]. The pulse generator connected to the electrodes is located under the skin at one’s waist. The size of an implantable pulse generator is similar to that of a pacemaker. The implantable pulse generator uses either a rechargeable battery, recharged by inductive coupling with an external module, or a long-lasting battery. External pulse generators can be worn around the waist and transmit the stimulation pulse through skin. An internal antenna picks up the signals and converts the electromagnetic signals to stimulating currents [19]. Neurostimulation was once considered as the last resort for pain treatment but more clinicians now are recommending it instead of chemical treatment. Many patients reported reduction or discontinuation of narcotic consumption in a recent 20 year literature review [20]. Six types of pain were reviewed. For back and leg pain, 616 patients were involved in 16 studies. With follow-up periods from 6 months to 5 years, pain outcome statistics indicate 56 % to 88 % reduction in pain with spinal cord stimulation and 52 % patients reported reduction or discontinuation of narcotic consumption. For complex regional pain syndrome, pain outcome for 260 patients in 12 studies during 6 month to 5 year periods indicates 57 % to 100 % reduction of pain with spinal cord stimulation. Fully 80 % of patients reported reduction or discontinuation of narcotic consumption.

### 5.2 Economic Significance

Current FDA approved neurostimulation applications include chronic pain management, Parkinson’s disease deep brain stimulation, and incontinence control. In addition, neurostimulation applications and markets are growing rapidly and significantly due to demonstrated effectiveness in new clinical uses. Dr. Jake Vander Zanden’s testimony before the Subcommittee on Health of the House Energy and Commerce on December 8, 2005, indicated that 25 % of Americans suffer from chronic pain, with 40 million physician visits per year, $50 billion is lost due to workday loss and $100 billion in medical expenses are due to chronic pain [21]. Business Communications Company reported that the market for pain management, comprised mainly of pharmaceuticals and devices, was $18 billion in 2000, growing at an average annual rate of 12 % to $32 billion in 2005 [22]. For deep brain stimulator applications, it is estimated that 60,000 new cases of Parkinson's disease are diagnosed each year, adding to the estimated 1.5 million Americans who currently have the disease. There were nearly 18,000 Parkinson’s disease-related deaths in U.S. in 2003 and the new patients’ ages are getting younger [23]. For incontinence control applications: it is estimated that 17 million Americans suffer from incontinence [24], [25]. More than two thirds of the patients are women. The total annual cost of care is estimated at more than $26 billion per year [26]. With even a relatively small portion of the patients with conditions mentioned above seeking neurostimulation as treatment of last resort, the economical impact is still massive. Overall, Medtech Insight in November 2006 estimated the total U.S. market for neurostimulation products, including cochlear implants, in 2005 was at $830 million. This market is expected to grow at a compound annual rate of 17.1 %, reaching more than $1.8 billion in 2010 [27]. The recovery to the losses in productivity and revenue due to the effects of neurodisorders to patients and their family could be much more significant [28], [29].

### 5.3 Technical Barrier and Potential Solutions

Impediment of technological and clinical progress in this area is due to the facts that neuroscience, neurology and neurophysiology are complicated science, animal test models are not well established, human tests are risky and time-consuming, and there is a lack of standardized means to define and characterize the effectiveness of neurostimulation. Neuron responses to pain are complex and difficult to predict depending on neurons probed, their locations, neuron types, physiological conditions, individuals’ health, environmental parameters, and pain sources. Lack of a deterministic correlation among pain stimuli, pain signals and neurostimulation effects makes quantitative documentation and evaluation difficult. The applications involve complex problems in both engineering (machine) and medical (human) aspects, so solutions need be addressed with intimate interaction and collaboration from both fields. New approaches and methods are required to define signals and parameters for pain and other neurodisorders. An effective way is to standardize definitions, technological requirements, medical/clinical requirements, measurement approaches and standards, instrumentation, and procedures to facilitate integrative collaboration. There are emerging needs for technology innovation in implant power sources, wireless communication, miniaturization, safe implant electrodes, recorders, and biocompatibility, as well as for clinical investigation in surgical apparatus, procedures, training, and characterization methods. These needs ought to be addressed coherently, integratively, and iteratively, especially for new applications currently in the research stage.

## 6. Patient-Centric Networked Medical Device Interoperability

### 6.1 Technological Innovation at Stake

Unlike the connected “plug-and-play” world of modern networked computers and consumer electronics, most medical devices are designed to operate independently, and do not employ open information and communication technology (ICT) networking standards for data communication or for device control. The integration of individual medical devices (such as physiologic monitors and medication delivery systems) into networked systems can provide comprehensive connectivity to the electronic medical record and can support advanced capabilities such as automated system readiness assessment, physiologic closed loop control of medication delivery, ventilation, and fluid delivery, decision support, safety interlocks, monitoring of device performance, plug-and-play modularity to support “hot swapping” of modules, selection of “best of breed” components, integration of surgical robot activities with other surgical and physiological events, and avoidance of unnecessary redundancy by using shared resources. Medical device vendors have not adopted cross-vendor standards-based interoperability for medical device communication. Therefore, when device integration is required, customized device interfaces must be developed, increasing costs and development time. Patient-centric medical device interoperability would benefit clinical areas as diverse as the operating room, intensive care unit, out-of-hospital transport, and general hospital clinical and ward care.

### 6.2 Economic Significance

As documented in *To Err is Human: Building A Safer Health System* [30], “more people die in a given year as a result of medical errors than from motor vehicle accidents, breast cancer, or AIDS, with total national costs (lost income, lost household production, disability and health care costs) of between $17 billion and $29 billion.” The potential benefits of applying modern engineering solutions, especially information and communications technology (ICT) intensive tools to these problems was outlined in a subsequent report entitled *Building a Better Delivery System: A New Engineering/Health Care Partnership* [31]. Kaiser Permanente recently presented a financial analysis of deploying comprehensive medical device-electronic medical record (EMR) connectivity with or without standards based ICT solutions. Kaiser projects that the cost of EMR integration adds 40 % to the cost of medical device acquisition, and that adoption of standards-based medical device connectivity will reduce Kaiser’s implementation costs by 30 % or approximately $12 million annually for the next 10 years [32].

### 6.3 Technical Barrier and Potential Solutions

Key barriers include the absence of vetted standards for medical data communication and control, a suitable plug-and-play system architecture, and the absence of requirements for an integrated clinical environment “ecosystem” that would include ancillary system functions such as data logging, data security, and device authorization, to provide a complete systems solution. The architecture must enable devices to function autonomously in a safe manner and support the deployment of smart alarms, clinical decision support, closed-loop control, enhanced diagnostics, reconfigurability, semantic interoperability, and allow implementation using currently available technology.

The methodology for gathering information on clinical requirements must be refined to assure adequate representation of the interoperability problem-space. New measurement technology and testing protocols are needed to assess the suitability of proposed standards, plug-and-play architecture, and ancillary devices to meet clinical requirements, including conformance to privacy and security requirements mandated by the Health Insurance Portability and Accountability Act (HIPAA). Interoperability and conformance testing tools will be needed to assess compliance of new devices to proposed solutions.

Over the past two years, groups of diverse stakeholders (clinicians, biomedical and clinical engineers, healthcare delivery systems, regulatory agencies, medical device vendors, and standards development organizations) have been convened to learn from past efforts in achieving medical device interoperability and to elicit clinical scenarios [33]. This body of work can inform the next stages of plug-and-play architecture development, and the development of assessment tools to match requirements and assess performance.

## 7. Summary

This report examined metrology and standards needs of medical devices used for computer assisted navigation and surgery, surgical robots (mostly manual control mode), surgical robots and phantom (artifact) devices, stimulation devices and drug-delivery and physiologic monitoring devices. For each category of these devices, the technological innovation at stake, economic significance, technical barrier and potential solutions were discussed. It is obvious from this discussion that these devices are of great significance for the health care delivery system, represent a very large market, and have several metrology and standard needs. Addressing these needs could unleash a great economic and therapeutic potential that will benefit the U.S. economy and welfare of millions of people.

Experiences with the computer devices and optical communications markets have proven that the early establishment of traceable metrology techniques and widely accepted standards has helped reduce cost and accelerate the acceptance and growth of these markets. Due to the health risks associated with the use of medical devices and their worldwide use, it is important that the problems identified in this paper are addressed by a coalition of medical professionals, metrologists, and international standards writing professionals in close collaboration with representatives from medical devices licensing and regulatory agencies worldwide.
